# Emotions of COVID-19: Content Analysis of Self-Reported Information Using Artificial Intelligence

**DOI:** 10.2196/27341

**Published:** 2021-04-30

**Authors:** Achini Adikari, Rashmika Nawaratne, Daswin De Silva, Sajani Ranasinghe, Oshadi Alahakoon, Damminda Alahakoon

**Affiliations:** 1 Research Centre for Data Analytics and Cognition La Trobe University Melbourne Australia; 2 College of Engineering and Science Victoria University Melbourne Australia

**Keywords:** COVID-19, pandemic, lockdown, human emotions, affective computing, human-centric artificial intelligence, artificial intelligence, AI, machine learning, natural language processing, language modeling, infodemiology, infoveillance

## Abstract

**Background:**

The COVID-19 pandemic has disrupted human societies around the world. This public health emergency was followed by a significant loss of human life; the ensuing social restrictions led to loss of employment, lack of interactions, and burgeoning psychological distress. As physical distancing regulations were introduced to manage outbreaks, individuals, groups, and communities engaged extensively on social media to express their thoughts and emotions. This internet-mediated communication of self-reported information encapsulates the emotional health and mental well-being of all individuals impacted by the pandemic.

**Objective:**

This research aims to investigate the human emotions related to the COVID-19 pandemic expressed on social media over time, using an artificial intelligence (AI) framework.

**Methods:**

Our study explores emotion classifications, intensities, transitions, and profiles, as well as alignment to key themes and topics, across the four stages of the pandemic: declaration of a global health crisis (ie, prepandemic), the first lockdown, easing of restrictions, and the second lockdown. This study employs an AI framework comprised of natural language processing, word embeddings, Markov models, and the growing self-organizing map algorithm, which are collectively used to investigate social media conversations. The investigation was carried out using 73,000 public Twitter conversations posted by users in Australia from January to September 2020.

**Results:**

The outcomes of this study enabled us to analyze and visualize different emotions and related concerns that were expressed and reflected on social media during the COVID-19 pandemic, which could be used to gain insights into citizens’ mental health. First, the topic analysis showed the diverse as well as common concerns people had expressed during the four stages of the pandemic. It was noted that personal-level concerns expressed on social media had escalated to broader concerns over time. Second, the emotion intensity and emotion state transitions showed that *fear* and *sadness* emotions were more prominently expressed at first; however, emotions transitioned into *anger* and *disgust* over time. Negative emotions, except for *sadness*, were significantly higher (*P*<.05) in the second lockdown, showing increased frustration. Temporal emotion analysis was conducted by modeling the emotion state changes across the four stages of the pandemic, which demonstrated how different emotions emerged and shifted over time. Third, the concerns expressed by social media users were categorized into profiles, where differences could be seen between the first and second lockdown profiles.

**Conclusions:**

This study showed that the diverse emotions and concerns that were expressed and recorded on social media during the COVID-19 pandemic reflected the mental health of the general public. While this study established the use of social media to discover informed insights during a time when physical communication was impossible, the outcomes could also contribute toward postpandemic recovery and understanding psychological impact via emotion changes, and they could potentially inform health care decision making. This study exploited AI and social media to enhance our understanding of human behaviors in global emergencies, which could lead to improved planning and policy making for future crises.

## Introduction

### Overview

The COVID-19 pandemic continues to devastate the world, with more than 80 million infections and more than 1.7 million deaths [1]. It is an unprecedented public health emergency that has prompted most governments to enforce hard borders, strict social distancing, and rigorous quarantine restrictions. The impact and aftermath of these public health emergency measures have resulted in a severe psychological burden for all individuals. The economic and social fallout has affected individuals and communities alike, resulting in mental health disorders and emotional distress that adds to already overwhelmed health care systems and services worldwide [2-4]. Although researchers and authorities are readily investing in vaccinations, social distancing regulations, and health care facilities to eliminate the virus, long-term mental health impacts will also require undivided attention and action to minimize the detrimental effects. Given this context, developing an understanding of the psychological and emotional burden will aid and accelerate postpandemic recovery and enable policy making for future emergencies of this scale.

Social restrictions and physical distancing measures during this pandemic have led to increased use of social media as a medium of communication by individuals and communities [5]. Expressions on social media during such crises are critical and representative of public opinion, as it has become the primary medium of communication for the exchange of information, experiences, and emotions with others who are facing similar challenges [6-8]. Identification of such opinions in the form of topics enables identification of people’s past and ongoing concerns throughout the pandemic [9]. Recent studies have shown a rapid growth of social media content focused on informational and emotional sharing as means of emotion regulation, avoiding mental health issues, and adjustments to the quality of life in lockdown [10,11]. This phenomenon has been described as the *social sharing of emotion*, which postulates that individuals who experience emotions are often eager to share and talk about their emotions [12]. Crises similar to the current pandemic may lead to the amplification of expression of emotions within a community or group of people [13-15]. The extent and frequency of sharing depend on the intensity of the emotional episode, and such social sharing occurs as a means of regulating one’s emotions. Thereby, the social sharing of emotions during a crisis creates a spreading and escalation of emotions within the group or community impacted by the event, which, in turn, reflect the mental health status of the community.

Given the volume and variety of content being shared on social media, online behaviors have been investigated as a *proxy* for offline human behaviors, with several studies reporting conclusive results and outcomes [16-18]. A massive number of social media conversations represents a cross-section of society that encompasses people’s opinions across different demographic dimensions. These emotions and opinions create a pool of untapped self-reported information. Therefore, it is relevant and useful to study the emotions and concerns voiced over such internet-mediated communication to better understand the mental health and emotional well-being during a time of crisis. Several recent studies have investigated the use of social media during the COVID-19 pandemic to discover topics, emotions, and sentiments from social media conversations [19-21] as well as different information sharing behaviors [22]. While the use of social media to understand public opinion is well established, studies suggest that more focus should be given to showcase how social media can be used to improve health knowledge [23].

On this premise, this study aims to investigate concerns and emotions expressed over social media to infer insights on people’s mental health as the pandemic progresses. It has been shown that emotions and concerns expressed on social media represent and relate to people’s underlying mental health [24-27]. This establishes social media as a lens through which to comprehend people’s mental health during a constrained situation such as the current pandemic, where conducting clinical trials is challenging.

We selected Twitter as the test bed social media platform for our experiments, given that it provides fast-paced, frequent, current affairs–focused end-user engagement, in comparison to other social media platforms [28]. We extracted tweets related to the pandemic that were posted from January to September 2020 and specifically focused on an Australian context based on the following reasons. First, Australia was one of the countries where distinct phases of the lockdown and their impact were clearly visible. This enables the temporal analysis of emotions and concerns in each phase to gauge insights into how citizens react and change their emotion-related behaviors as the pandemic progresses. The four phases are (1) prepandemic—when the outbreaks were yet to be declared a global pandemic and no positive cases have been reported in Australia, (2) the first lockdown—social restrictions following the first wave of positive cases locally, (3) easing restrictions—relaxation of social restrictions when the case numbers were brought under control, and (4) the second lockdown—following the emergence of new cases. Next, as Australia is one of the few countries with a sizeable population to successfully suppress both first and second waves of the pandemic [29,30], it was pertinent to study the emotions related to COVID-19 as experienced by the Australian public. Despite the successful management, a recent study mentions that mental health problems were widespread among Australians during the lockdown periods, where one-quarter of the participants in the study showcased mild to moderate symptoms of anxiety or depression [31,32]. The use of social media among the Australian public is well established for being representative of a broad cross-section of society [33-35], especially during emergencies as a powerful communication tool [36,37]. This allows us to investigate the emotions expressed via virtual platforms in order to determine similar behaviors related to Australian citizens’ mental health.

In this study, we focus on the application of artificial intelligence (AI) approaches that have been validated across several studies [18,27,38] to analyze human emotions. The algorithms are used to quantify emotion intensities, detect emotion state transitions, and identify profiles of impacted individuals based on the concerns they have expressed on social media to provide a holistic view of people’s mental health. This approach can be described as an ensemble of machine learning algorithms and natural language understanding (NLU) techniques that establish an end-to-end pipeline to extract, process, normalize, analyze, and aggregate self-reported information in its unstructured format into topics and emotions expressed during the key phases of the pandemic. We have also compared the two lockdown periods and the social media expressions in response to restrictions imposed during each lockdown to capture changes in temporal emotions and related concerns.

The rest of the paper is organized as follows. The Methods section presents an overview of the methodology used in this study and the Results section presents the outcomes of the analysis. The paper concludes with the Discussion section, which outlines the implications of the study, limitations, and potential avenues for future research.

### Background

The current literature reports many research studies that detect sentiment and emotions from social media using a variety of techniques. Sentiment analysis has been explored using lexical and statistical approaches [39-42] and recent research studies have extended this by using advanced deep learning models that can detect the bipolarity of social media conversations [43-46]. While sentiment analysis classifies posts into negative and positive categories, emotion detection extends this categorization by detecting granular variants of emotions [47]. Many emotion models, such as Ekman’s basic emotion model [48], the valence and arousal model [49], and Plutchik’s emotion model [50], are being used as the basis for emotion detection. Most of the approaches of emotion detection rely on prelabeled data via crowdsourced annotations and semantics to develop machine learning models [51-53]. Current research studies report the use of many deep learning models, such as word embedding models [46,54], convolutional neural networks [55], and recurrent neural networks [56-58], for emotion classification. Emoticons and emojis have also been used in studies to infer emotions from social media as they represent user-annotated labels for emotion classification [56,59]. In addition to the emotion classification, emotion intensity is also captured via the annotated data sets [52]. A more recent research study reports on the use of the stacked ensemble method for emotion intensity detection that enables the identification of expressions that denote the emotion intensity [60].

However, these approaches are focused on detecting sentiment or emotion from a social media post but do not focus on emotion modeling, which makes it possible to explore underlying changes of intensity, shifts, and patterns of change in emotions and to generate insights by linking to associated human behaviors [61]. The lack of labeled data for emotion detection is also a major drawback in using supervised machine learning approaches. Therefore, it requires a robust method to explore emotions in unstructured, voluminous social media data that does not require prior knowledge about the data. In this study, we use an ensemble of word embedding and NLU techniques to derive emotions from unlabeled social media conversations. As the foundation of the emotion analysis, we use Plutchik’s model [50], which comprises eight basic emotions: anger, sadness, joy, trust, anticipation, fear, disgust, and surprise. The emotion classification is based on these eight emotions, and their intensities are mapped to show the strength of each emotion.

## Methods

### Data

Twitter conversations that were posted from January to September 2020 within Australia or an Australian context were extracted. Popular hashtags related to COVID-19 in Australia were used to query the data sources. Content by news channels and bots was identified by examining an unusual volume of data generated by each user and was eliminated from the analysis. This process yielded 73,000 public conversations by Twitter users in Australia. The extracted data were cleaned, preprocessed, and anonymized before being used in the analysis.

In order to identify different phases of the pandemic, the stages from Table 1 were formed to align with the timeline of the pandemic experience in Australia [62].

**Figure 1:**
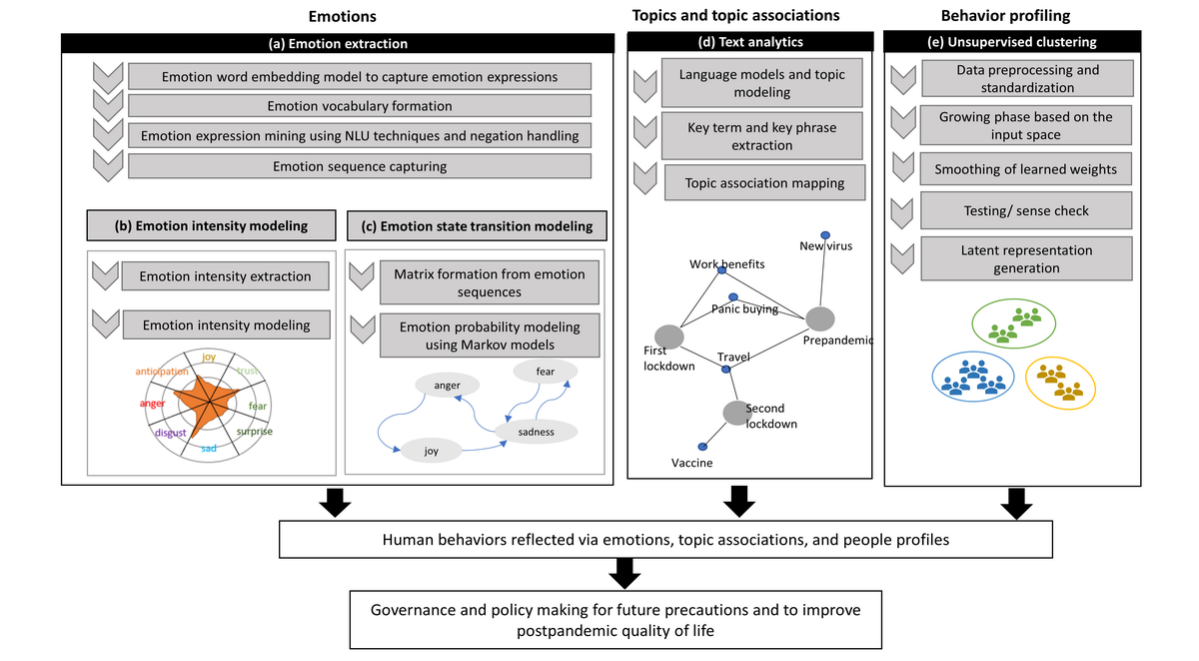
The high-level view and workflow of the artificial intelligence framework. NLU: natural language understanding.

Figure 1 presents the high-level view and the workflow of the framework with the respective components.

**Table 1:** The four main phases of the COVID-19 pandemic from January to September 2020.

Time period (in 2020)	Phase
January to February	Prepandemic
March to May	First lockdown
June	Easing restrictions
July to September	Second lockdown

### Emotion Analysis

The emotion extraction, intensity generation, and emotion transition modeling process is shown in the emotions component of the architecture diagram in Figure 1 (a). The emotion analysis was conducted to represent the emotions of each stage as well as the temporal changes in emotions over time. The emotion extraction was carried out on chronologically ordered social media conversations in order to represent emotions over a conversation as a sequence of individual, single-post emotions. First, we trained a word2vec [63] word embedding model, which is a deep learning technique to create vector representations of textual data. This allows the positioning of semantically similar terms together, thereby enabling the finding of closely associated terms for each basic emotion. This querying process yielded a rich vocabulary for representing each emotion, from the basic emotions, which was used as the basis for emotion extraction.

Next, emotion negation was handled to fine-tune the extracted emotions. This process resulted in a sequence of emotions that denoted the chronologically ordered emotions and change of emotions over a conversation. The intensity of emotions in the four stages was used to model an intensity profile for each stage. We have used a modified intensity-capturing algorithm presented in Adikari et al [16] that accommodates the frequency and the presence of expressions that increase or inhibit the valence of the emotion. This allowed for the identification of prominent emotions and their differences in each stage, as seen in Figure 1 (b).

The temporal emotion analysis was conducted by modeling emotion transition models, which models the probabilities of emotion state changes over a course of time. Affective computing research suggests that basic emotions at a particular instance—represented by one post of a tweet in this application—can be regarded as discrete states, and the interactions among these can be modeled to represent the likelihood of the change in the state of emotions [64]. For this purpose, a mathematical model can be used to model the interactions between the emotion states. We have used the sequence of emotions at each stage to generate respective emotion transition models using Markov models [65,66], as seen in Figure 1 (c). The use of emotion transitions to capture individual and group emotions using social media conversations has been successful in a smart city context [17] and for analysis of mental health via online data sources [16]. We have applied this to model the emotion state transitions of each stage, which reflect the flow of emotions as the pandemic progressed.

### Topic and Topic Association Analysis

The topic analysis was conducted based on a series of NLU techniques to capture topics of discussions from social media data, as seen in Figure 1 (d). The topics represent people’s concerns and, therefore, encompass useful insights on their discourse patterns related to COVID-19 concerns. Outcomes of this analysis help in better interpreting the outcomes of the emotion analysis by providing contextual information for different emotion changes.

To conduct the topic analysis, the data set was first cleaned and processed in order to remove weblinks, stop words, news links, and repeating content generated by bots. This cleaned data set was then split into each stage and taken for the topic analysis. Given the large amount of content, an unsupervised topic modeling technique was used, which groups similar terms based on latent Dirichlet allocation [67]. Once the prominent topic clusters were identified, a trained word2vec embedding model and an automated keyphrase extraction algorithm [68] were used to identify similar topics and subtopics. The outcomes generated the most frequently mentioned terms as well as semantically prominent terms, which allowed us to understand a discrete topic from the terms. The analysis was conducted separately for the four stages to understand which topics were prominent in each stage; thereafter, a topic association map was created to identify common topics across stages to demonstrate people’s continuing concerns.

### Behavior Profiling

Behavior profiling was conducted to identify different groupings of citizens in the community based on their emotions and concerns expressed over social media. Identification of such profiles can form a visualization of different profiles that exist in society. This representation is useful to better understand different citizen needs and can inform decision making for relevant authorities.

For profiling, we used an improved variant of the growing self-organizing map (GSOM) algorithm [69]. The GSOM presents a map topography that self-structures by adapting its size and shape based on the attributes and variations of input data. The GSOM algorithm utilizes competition and correlative learning based on the data provided. As input data are presented, nodes of the network compete among each other for ownership of the input, and the winners strengthen their relationships with this input. The competitive learning process is repeated for the complete data set for several cycles, and ultimately the map associates output nodes with patterns in the input data set. The GSOM acts as a knowledge map and can discard outdated information and overfitting of knowledge during the knowledge acquisition process [70,71] that allows the retention of relevant information only. The primary reason to incorporate such mature self-structuring AI was to discover hidden representations of behavior profiles based on their discourse and emotion patterns, which is otherwise challenging to interpret from a large amount of data. The ability of the GSOM algorithm to uncover patterns among data without prior training or supervision makes it possible to detect different groups of behaviors present among the citizens.

### Ethics Statement

We have obtained ethics approval for this research from the La Trobe University Human Ethics Committee.

## Results

### Topics and Topic Associations

**Figure 2:**
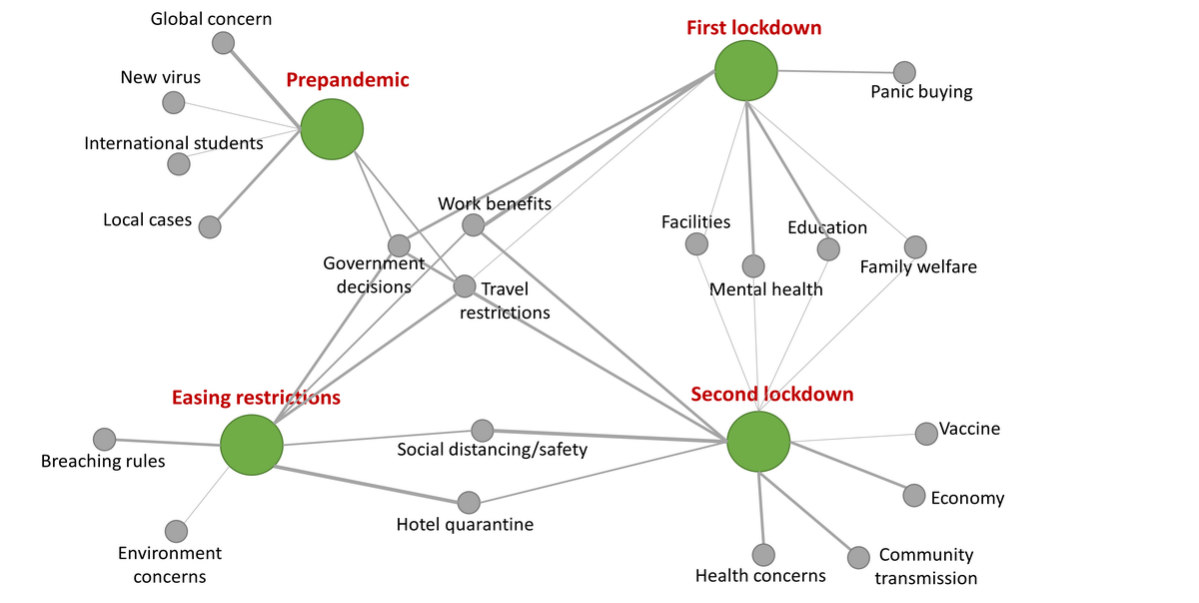
Topic associations between the four stages of the pandemic.

Social media contains a high volume of expressions by individuals and groups alike. However, most of these expressions can be aggregated into a finite number of themes and topics [72]. We conducted a topic analysis and a topic association analysis to explore the distinct and common themes across the four stages of the pandemic. Table 2 presents the topics discussed during the four stages of the pandemic, from January to September 2020. The most prominent topics in each stage are presented in Table 2, where the quantitative measure indicates the weight of each topic based on the volume. The topics were also associated across stages as depicted in Figure 2. Key observations are that *government decisions* and *travel restrictions* were discussed during all four stages, and both lockdown stages expressed similar topics oriented toward lifestyle factors. These common topics included *education*, *mental health*, *family welfare*, and *facilities*. *Panic buying* was only mentioned during the first lockdown and was entirely absent during the second lockdown. During the easing restrictions stage, people mostly talked about *social distancing and safety* as well as *hotel quarantine*, which were common topics during the second lockdown as well. Apart from the common topics, the second lockdown mainly focused on *health care*, *community transmission*, *economy*, and *vaccine*. These denote that the conversations were more informed and regulated by the second lockdown. It should be noted that only conversations with prominent topics were included in the analysis and that general conversations have been omitted from the topic analysis. The outcomes of this analysis also aid in comprehending the emotions by providing contextual information related to each stage.

**Table 2:** Topics of discussion across the four stages of the pandemic.

Pandemic stage and main themes			Volume of conversations, n (%)
**Prepandemic (n=15,302)**			
	Global concern	6021 (39.35)	
	Local cases	3522 (23.02)	
	Travel restrictions	2518 (16.46)	
	Government decisions	2127 (13.90)	
	International students	588 (3.84)	
	New virus	526 (3.44)	
**First lockdown (n=23,201)**			
	Work benefits	6116 (26.36)	
	Education	5832 (25.14)	
	Government decisions	3765 (16.23)	
	Travel restrictions	3069 (13.23)	
	Mental health	2568 (11.07)	
	Family welfare	1176 (5.07)	
	Facilities	413 (1.78)	
	Panic buying	262 (1.13)	
**Easing restrictions (n=11,801)**			
	Government decisions	2811 (23.82)	
	Travel restrictions	2700 (22.88)	
	Hotel quarantine	2294 (19.44)	
	Breaching rules	1554 (13.17)	
	Work benefits	1184 (10.03)	
	Social distancing and safety	925 (7.84)	
	Environment concerns	333 (2.82)	
**Second lockdown (n=16,198)**			
	Social distancing and safety	2910 (17.96)	
	Health care	2112 (13.04)	
	Work benefits	2046 (12.63)	
	Government decisions	1902 (11.74)	
	Community transmission	1793 (11.07)	
	Facilities	897 (5.54)	
	Economy	886 (5.47)	
	Hotel quarantine	867 (5.35)	
	Education	841 (5.19)	
	Family welfare	829 (5.12)	
	Mental health	489 (3.02)	
	Vaccine	381 (2.35)	
	Travel restrictions	245 (1.51)	

### Emotion Classification and Intensities

The AI approach used for the study of emotions expressed during the pandemic began with the classification of emotions, based on the widely cited psychological emotion model proposed by Plutchik [50]. Classification of emotions was followed by the quantification of the intensity of each of the eight emotions as they were expressed during the four stages.

The outcomes of the emotion analysis suggested that both *sadness* and *fear* emotions were the most intense during the prepandemic stages. This is indicative of the central emotional response during a pandemic being *fear* [2]. In addition, it is said that given the instinctive defensive systems for combating ecological threats, humans experience negative emotions resulting from a threat that can be contagious. The high intensity of *sadness* and *fear* identified during the announcement stage of the pandemic exhibits this emotional pattern. This behavior was also confirmed by the emotion transition model in this stage, where higher probabilities were observed for these emotions. Furthermore, *fear* is also associated with *panic*, where people act blindly and excessively out of self-preservation, possibly endangering the survival of others [2]; this was experienced via incidents like *panic buying*, where people unreasonably stocked up on essential items for impending self-isolation. The surfacing of fear and sadness emotions represents increased anxiety, which impacts people’s mental health [73].

The temporal emotion analysis showed that, out of the negative emotions, *anger* and *disgust* were more strongly expressed toward the latter part of the pandemic due to the security breaches and restrictions. Terms such as “covidiot,” “quarantine issues,” “restrictions,” and “another lockdown” were mostly mentioned in the Twitter conversations related to these emotions. This indicates that starting from the negativity of fear and sadness, public emotions have transferred to more anger and disgust at a later stage.

**Figure 3:**
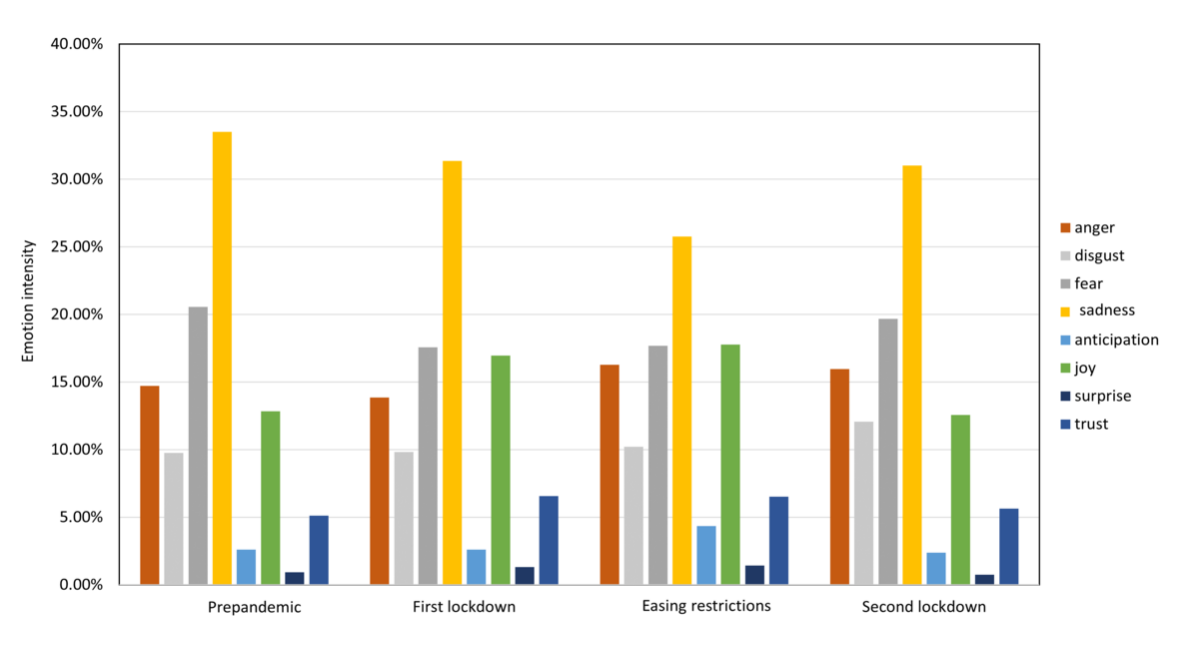
Emotion intensity fluctuations over time.

Positive emotion expressions were also captured by the analysis. It was noted that *joy* was more strongly expressed during the easing restriction stage and least during the second lockdown stage. This behavior aligned with real-world behaviors, as it was expected that people would express disappointment for having to experience another lockdown. The emotions *trust* and *anticipation* demonstrated higher intensities during the first lockdown and easing restriction stages; however, they have subsided over time (Figure 3).

We conducted a comparative study based on the two lockdown stages to identify the differences in emotion intensity (Table 3). The differences were compared using the Pearson chi-square test [74,75], and the confidence intervals were calculated based on the recommended method given by Altman et al [76]. A significance level of *P*<.05 was used to determine if the differences in the intensities were significant.

The comparison of negative emotions shows that, except for *sadness*, all other negative emotion intensities were significantly higher in the second lockdown (*P*<.05). This evidently demonstrates the increased disappointment and negativity of having to experience a second lockdown. The emotion *sadness* was persistent in both lockdowns.

The positive emotion comparison shows that, apart from *anticipation*, all other positive emotions were strongly expressed in the first lockdown (*P*<.05). This suggests that people had elicited more positive thoughts during the first lockdown compared to the second. The increase in the intensity of negative emotions and the reduction of positive emotions with further lockdowns confirm the increased levels of distress and deterioration of mental health. Based on this, relevant strategies should be initiated in order to eliminate the spread of negativity among people in further lockdowns.

**Table 3:** Comparison of emotion intensities between the first and second lockdowns.

Emotion		Normalized emotion intensity during the first lockdown (n=36,317), %	Normalized emotion intensity during the second lockdown (n=10,604), %	Difference, % (95% CI)	*P* value
**Negative emotions**					
	Anger	13.84	15.97	2.13 (1.3567 to 2.9218)	<.001
	Sadness	31.36	31.01	0.35 (–0.6563 to 1.3461)	.49
	Disgust	9.81	12.06	2.25 (1.5691 to 2.9518)	<.001
	Fear	17.58	19.66	2.08 (1.2365 to 2.9399)	<.001
**Positive emotions**					
	Joy	16.94	12.56	4.38 (3.6308 to 5.1097)	<.001
	Surprise	1.32	0.74	0.58 (0.3662 to 0.7708)	<.001
	Trust	6.56	5.63	0.93 (0.4108 to 1.4260)	<.001
	Anticipation	2.59	2.37	0.22 (–0.1257 to 0.5406)	.21

### Emotion Transitions

**Figure 4:**
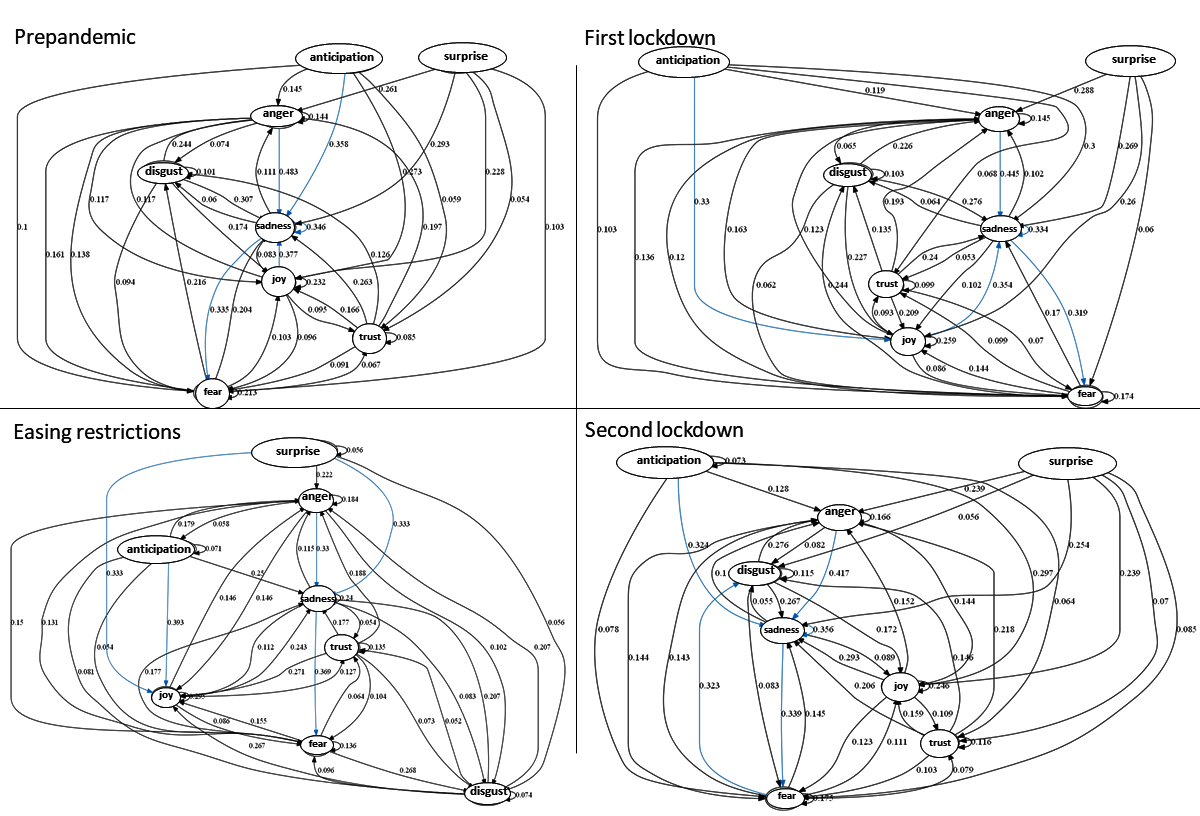
Emotion transitions of the four stages of the pandemic. Each arrow represents a transferring of one emotional state to another. The numbers on the arrows represent the likelihoods (ie, probabilities) of changed emotion states.

Mapping and quantifying the transition of emotions over conversations can be useful to predict the likelihood of emotion state changes as well as to observe temporal change in emotions and intensities over time. In a social media setting, this represents collective emotions in a community over time. The emotion state transitions were modeled separately for each stage, which enabled temporal analysis of emotions from the prepandemic stage to the second lockdown stage. For generating emotion transitions, the emotion space was defined as a set of discrete emotions—anger, fear, sadness, disgust, joy, anticipation, trust, and surprise*—*from the emotion extraction described earlier. Based on the temporal emotion sequence extracted from the emotion extraction module, a matrix was formed with the frequency of transitions of emotions from one state to another, and the matrix was used to create the emotion state transition diagrams shown in Figure 4; the shift and emergence of emotions are demonstrated via the arrows connecting the emotion states. The probabilities denote the likelihood of changed emotion states (Table 4).

Transitions at each stage demonstrate the temporal analysis of emotions during the pandemic. This emotion flow is useful when determining how people’s emotions have changed over time. Health care practitioners and the government can use this information and analysis to understand the mental health status of the population.

Regarding the outcomes, it was observed that most of the emotion transitions involved the *sadness* state in the prepandemic stage. *Sadness to fear* was also prominent relative to other emotion changes. By the first lockdown, there was a shift toward positive emotions, as the emotion states *anticipation* and *joy* were seen to be prominent. This pattern deviated slightly in the third stage, where positive emotions had amplified and negative emotions had reduced. The emotion propagation made a drastic change in the second lockdown, where positive emotion behaviors had decreased and negative emotions had been amplified, eliciting more tendencies toward *sadness*, *fear*, and *disgust*. This propagation of emotions denoted the emotion flow among people as they voiced their thoughts on social media.

The emergence and shift of emotions over different points in time were seen to be associated with the different stages across different time points in the COVID-19 pandemic. It is apparent that people experienced diverse emotions and had expressed them on social media reflecting their individual interests, opinions, and priorities. Further analysis was conducted using profiling techniques to investigate the intensity (ie, valence) of these emotions and the variation across different stages, which provided insights into who these people were and their priorities and opinions during the pandemic.

**Table 4:** Top five emotion transitions and probabilities for each pandemic stage.

Emotion transition		Probability
**Prepandemic**		
	Anger → sadness	0.483
	Joy → sadness	0.378
	Anticipation → sadness	0.358
	Sadness → sadness	0.346
	Sadness → fear	0.335
**First lockdown**		
	Anger → sadness	0.445
	Joy → sadness	0.354
	Sadness → sadness	0.334
	Anticipation → joy	0.329
	Sadness → anticipation	0.288
**Easing restrictions**		
	Anticipation → joy	0.393
	Surprise → joy	0.389
	Sadness → fear	0.362
	Anger → sadness	0.340
	Joy → joy	0.295
**Second lockdown**		
	Anger → sadness	0.417
	Sadness → sadness	0.357
	Sadness → fear	0.341
	Anticipation → sadness	0.324
	Fear → disgust	0.322

### Group Profiles Based on Topics and Emotions

**Figure 5:**
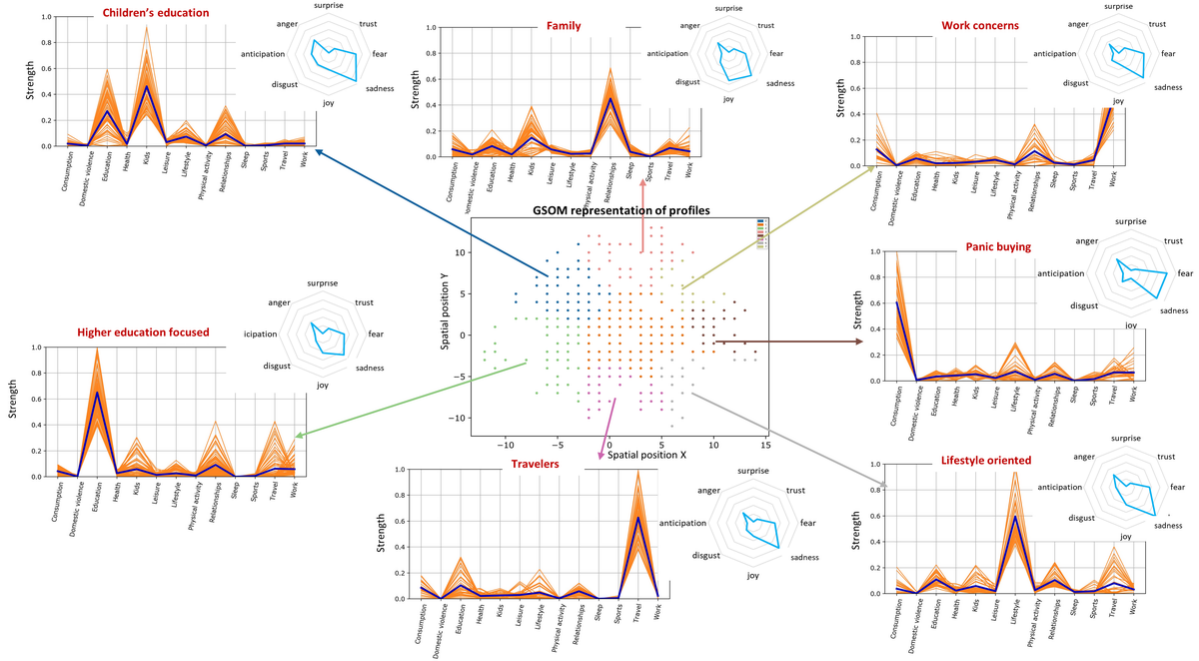
Behavior profiles in the first lockdown. The mean strength of each concern is denoted as a blue line. GSOM: growing self-organizing map.

**Figure 6:**
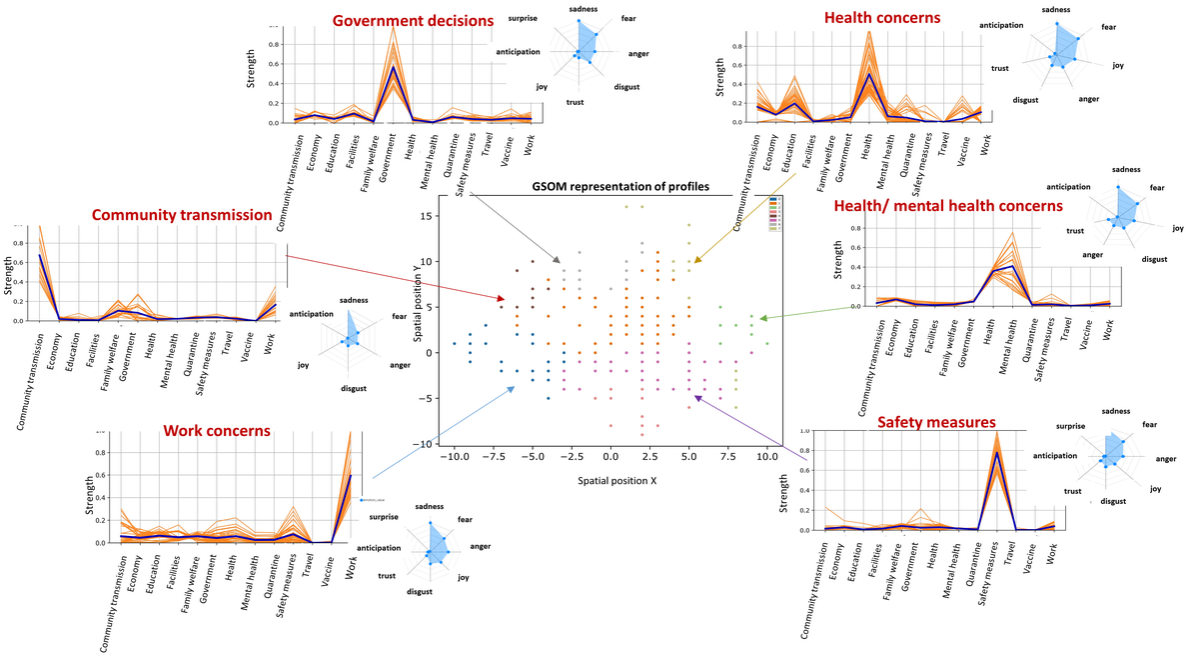
Behavior profiles in the second lockdown. The mean strength of each concern is denoted as a blue line. GSOM: growing self-organizing map.

The GSOM algorithm was used to generate profiles of individuals based on topics and emotions. This enabled the identification of different clusters of citizens that existed within the community in terms of their concerns and emotions. Figures 5 and 6 illustrate the profiles identified for the period of the first and second lockdowns, respectively. Seven profiles were identified for the first lockdown based on the topics and emotions expressed by individuals, and they were labeled based on the most prominent topics that have been discussed. The profiles were (1) children’s education, (2) family-oriented discussions, (3) work concerns, (4) panic buying, (5) lifestyle-oriented discussions, (6) travelers and traveling, and (7) higher education–focused discussions.

These profiles demonstrated a distinct focus in their conversations, which resulted in grouping those individuals together. Among these profiles, the *panic buying* cluster demonstrated higher levels of *fear*, whereas *sadness* was prominent in all clusters. In addition, the *family* cluster demonstrated more *joy*, which can be aligned with spending more time with family.

The second lockdown profiles shifted slightly from the profiles identified in the first lockdown. The identified behavior profiles were (1) government decisions, (2) health and mental health concerns, (3) community transmission, (4) safety measures, and (5) work concerns.

Compared to the first lockdown, the profiles of the second lockdown exhibited more negative emotions, where increased levels of negative emotions can be seen in health and mental health concerns. It is noteworthy to observe that the profiles formed in the second lockdown were more focused toward broader aspects of the pandemic when compared to the more lifestyle-oriented profiles in the first lockdown. This difference indicates that as the pandemic progressed, people’s concerns shifted more toward broader aspects than concerns at the early stages, which may have been normalized with time.

## Discussion

### Principal Findings and Implications

At a high level, the themes and topics that were seen at the individual level in the first stages of the pandemic had cascaded into broader and informed topics during the second lockdown. Individuals frequently discussed matters related to family, panic buying, and facilities during the first stage and transitioned into generic topics, such as the vaccine and economy, in the latter stages. Besides, several common concerns persisted over the course of time. Identification of such behaviors related to topics and topic associations from social media conversations can inform public health authorities regarding necessities and the issues that disrupt people’s quality of life during this global pandemic. However, it is crucial to investigate the underlying emotions in these conversations to gauge the status of people’s mental health.

### Limitations and Future Work

One of the limitations of this study is the use of a single data source. As Twitter users are not representative of the whole of Australia, we believe that this could pose a limitation when modeling the emotions and concerns of the Australian population. However, given the importance of Twitter as a social media platform and the substantial amount of data retrieved (73,000 conversations), we were able to capture a broad cross-section of the community. As future work, the study could be extended by using multiple data sources, such as online forums and other social media channels. This will create a holistic view of the community. Moreover, the investigation could be conducted using data from another country, as this would provide different insights into how people have reacted toward different restriction measures and pandemic stages.

### Comparison With Prior Work

Our findings regarding emotions are consistent with and align with public health studies related to the COVID-19 pandemic. The emergence of the *sadness* and *fear* emotions during the initial stages resemble the increased anxiety and depression faced by Australians in the first few months of the pandemic [31,32]. Past research indicates that public health emergencies often trigger negative emotions, as people tend to develop aversion, anxiety, and fear [77,78]. The emotion *fear* is often associated with such emergencies and pandemics, as it is one of the core emotions linked with survival. It has been reported that the human mind’s instinctive defensive systems for combating ecological threats are often wired with negative emotions resulting from a threat that can be contagious [78]. Moreover, fear is also associated with panic behaviors, where humans act egoistically, endangering others’ lives as well. These behaviors were captured via the emotion and topic analysis of this study, demonstrating that social media reflected people’s underlying emotions and mental health concerns.

Studies investigating social media sentiments and topics during the pandemic align with our study [20,79,80]. In addition, our study provides an extension to the current body of COVID-19 social media research by modeling the emotion state transitions over time to represent the shift of emotions over time. The profiling of citizens based on emotions and concerns also enables the identification of clusters in the community during different stages of the pandemic. Recent research related to identifying the role of social media in the COVID-19 pandemic suggested further exploration of how social media can be utilized to inform health care practices [23]. We believe that our study contributes to providing outcomes that can enhance health care practices related to community understanding and uplifting of mental health care during a similar crisis.

### Conclusions

As the entire world is still grappling with the direct or indirect effects of the COVID-19 pandemic, it is pertinent that we explore and analyze human emotions and related expressions in order to aid postpandemic recovery, as well as to prepare for future crises. Given the current social isolation settings, people are experiencing increased mental health issues and emotion changes that are affecting their quality of life [81,82]. During times of social isolation, the abundance of data available in digital spaces provides a reflection of people’s behaviors, which is otherwise challenging to assess via clinical trials or interviews. This enables emotion analysis using digital spaces to act as an adjunct to real-world behavior analysis [83].

This study focused on capturing human emotions and concerns, which were reflected through social media conversations as the COVID-19 pandemic progressed. Our work is targeted at detecting and helping to understand the underlying mental health– and well-being–related issues via emotions expressed and topics discussed on social media by different segments of society. Although prevention of spread with social distancing and masking, as well as attempts at the elimination of COVID-19 with vaccine programs, have been widely discussed, less attention has been paid to the less visible issue of emotional distress and mental health. Governments and health care organizations are now realizing the deep and long-term effects of these issues; funding is being allocated to address these issues and programs for understanding the implications are now being established [81]. The work described here is focused on this less discussed area. We proposed an AI-based approach and designed and developed a supporting technical framework to learn about the emotion and content representations from unstructured, voluminous social media data, which are otherwise challenging to assess manually. The outcomes from this study aligned with theories of social sharing, emotional responses during pandemics, and collective emotion theories, and they represent insights that can be used to improve the mental health of citizens.

The outcomes of the emotion analysis demonstrated a high intensity of fear and sadness emotions during the announcement stage of the COVID-19 pandemic, and the emotion transition model also demonstrated higher probabilities of expressing fear and sadness during the prepandemic stage among Australian citizens. Noteworthy insights were produced as the outcomes of the temporal emotion analysis. The emotion transition models across the pandemic demonstrated how the emotions had changed over time. It was noted that, although fear and sadness emotions were more prominent over the first few months, they had eventually transitioned into anger and disgust as people expressed their dissatisfaction and frustration with the continuing pandemic and restrictions. The temporal emotion analysis during the pandemic using emotion transition models was one of the main contributions of this study, as it demonstrates how people’s emotions change over time. On the other hand, positive emotions such as trust and anticipation were observed in the first lockdown and easing restrictions stages; however, they had diminished over time as the pandemic progressed. This *emergence and shift* of different emotions is a strong indicator of people’s varying emotions, and it signals the underlying mental health of the population. The decreased intensity of sadness and fear in the latter stage showed the normalization of emotions as people tended to realize the nature of the pandemic. However, the increased emotion transitions toward anger and disgust as well as broader concerns represented the dissatisfaction toward the management of the pandemic. These emotion behaviors presented insights into people’s emotions, which were challenging to quantify and measure using traditional surveys or clinical trials, given the social isolation settings. Social media has been effective in helping us understand human emotion expressions during times of crisis, and it can be utilized to improve health care practices in similar crises.

On the other hand, emotions expressed on social media can be contagious, as negative content attracts more attention and propagation on social media [84,85]. The increased intensity of negative emotions and the persistence of negativity over time that was captured across different stages in this study aids this phenomenon. Negative emotions were observed in both first and second lockdowns and were significantly higher during the second lockdown. Therefore, it is crucial to observe this negative content to avoid further escalation. These swirls of emotions generated at one point in time have the ability to propagate through time using social media as the medium, as social media holds the potential for cascading and going viral to transmit emotional content more rapidly and broadly than other media [86,87]. These behaviors can also be aligned with the social sharing of emotion, which states that collective emotional events are anticipated to trigger a social process of emotion propagation [12]. It has been stated that emotion strength, presence, and valence can affect and influence citizen engagement through social media; therefore, it is imperative to moderate and monitor such emotional behaviors on digital platforms [88]. As potential implications, the proposed emotion behavior analysis in the study can be used to moderate online negativity and even detect distress among people during the pandemic to uplift their mental health as well as in postpandemic times as measures of precautions.

Another key observation of this study was the profiling of people based on similar emotions and thematic expressions on social media. It was observed that while certain groups of people were more enthused about family matters, some groups expressed more concerns related to employment issues. The deviation of profiles from the first lockdown to the second lockdown also shows how the identified profiles took different shapes depending on the concerns at that point in time. Such behavior profiling enables the understanding of different groups of people with different concerns and emotions. Such groups elicit significant insights and enable us to look at the community from different perspectives. Based on these behavior changes, governance and health care policy-making practices could be tailored to consider different groups of concerns emerging from different sectors within the community. Insights can also inform future planning to uplift citizens’ mental health during disastrous events similar to the current pandemic.

Apart from the aforementioned implications, one of the key outcomes of this study was to showcase the applicability and capability of using social media conversations to identify, analyze, and understand human behaviors at scale during a global crisis. Digital avatars created in social media have become a derivative of reality. This study showcases that in the age of social media, it is helpful, or even essential, to study human behaviors and emotions using their digital representations.

## Abbreviations

AI: artificial intelligence
GSOM: growing self-organizing map
NLU: natural language understanding
